# Influencing factors analysis of clinical effect of heart failure patients treated with ivabradine and metoprolol succinate and construction and validation of nomogram prediction model

**DOI:** 10.3389/fcvm.2025.1571468

**Published:** 2025-10-09

**Authors:** Guoxiang Wu, Daqiu Chen, Lifang Chen, Yanqing Wu, Suying Guan, Feng Wu, Yixing Chen, Xianhua Ye, Tao Yang

**Affiliations:** ^1^ Department of Cardiology, Nanping First Hospital Affiliated to Fujian Medical University, Nanping, China; ^2^ Department of Health, Nanping First Hospital Affiliated to Fujian Medical University, Nanping, China; ^3^ Department of Cardiology, The Affiliated People’s Hospital of Xinxiang Medical University, Xinxiang, China

**Keywords:** heart failure, ivabradine, metoprolol succinate, nomogram prediction model, clinical effect

## Abstract

**Objective:**

To analyze the influencing factors of the clinical effect of ivabradine (Ivab) combined with metoprolol succinate (Met-S) in patients with heart failure (HF), and to construct and verify the nomogram prediction model, in order to provide reference for clinical treatment.

**Methods:**

250 cases of HF patients from January 2021 to June 2023 were selected. The relevant factors affecting the therapeutic effect were screened out through univariate and multivariate analysis. The nomogram prediction model was constructed, and the model was verified and evaluated using receiver operating characteristic (ROC) curve, calibration curve and decision curve analysis (DCA).

**Results:**

Single factor and multiple factor analyses showed that LVEF, LVEDD, 6 MWT, heart rate and BNP level were the independent risk factors for clinical effects (*P* < 0.05). In the training and testing sets, the area under the ROC curves were 0.862 (95% CI: 0.776–0.947) and 0.819 (95% CI: 0.704–0.934), respectively. The calibration curve showed good consistency, and DCA analysis indicated that the model had clinical application value.

**Conclusion:**

LVEF, LVEDD, 6 MWT, heart rate and BNP level affect the clinical effect of Ivab combined with Met-S in patients with HF. The nomogram prediction model established has high accuracy and clinical application value.

## Introduction

Heart Failure (HF) is not a single disease, but the end stage of development of various heart diseases, and the pathological mechanism involves myocardial injury and cardiac overload (1, 2). The impaired myocardium cannot pump blood effectively, resulting in the difficulty of cardiac output to meet the metabolic needs of the body, and triggering a series of complex symptoms (3). Patients often present with dyspnea, which may only occur after activities in the early stage. As the disease progresses, episodes may occur in resting state, seriously affecting the quality of life (4, 5). Fluid retention causes edema of the lower limbs, which gradually spreads from the ankle to the whole body (6, 7). The persistent sense of fatigue limits the patients' ability to move daily (8). The morbidity and mortality of HF remain high, and its prevalence shows an increasing trend with the aggravation of population aging and the increase of risk factors for cardiovascular diseases (9).

At present, drug therapy is an important part of comprehensive treatment of HF (10, 11). As a novel sinoatrial node If current-specific inhibitor, Ivabradine can selectively reduce heart rate without affecting myocardial contractility and ventricular repolarization (12, 13). Metoprolol Succinate (Met-S) was a selective β1 receptor antagonist that can reduce heart rate, blood pressure and myocardial oxygen consumption and improve myocardial remodeling by inhibiting sympathetic nerve activity (14). The combination of ivabradine (Ivab) and Met-S, in theory, has a synergistic effect, and can more effectively control the symptoms of patients with HF and improve the prognosis.

However, in clinical practice, there may be a variety of factors that affect the clinical efficacy of this combination regimen. Accurately identifying these influencing factors is of great clinical significance for optimizing the treatment strategy of HF and improving the treatment effect. Therefore, the purpose of this study was to analyze the influencing factors of the clinical effects of Ivab combined with Met-S in patients with HF, and to construct and verify the nomogram prediction model, in order to provide more valuable reference for clinical treatment.

## Data and methods

### Study objects

With the approval of our Ethics Committee, we retrospectively analyzed data from HF patients who received Ivab combined with Met-S in our hospital from January 2021 to June 2023. Finally, 250 cases were included and randomly divided into a training set and a testing set according to the ratio of 7:3. The requirement for informed consent was waived by the Ethics Committee due to the retrospective nature of the study. Inclusion criteria: (1) Compliant with diagnostic criteria for HFrEF (LVEF ≤40%) per 2022 AHA/ACC/HFSA Guidelines (15); (2) Age ≥18 years; (3) Treatment with Ivabradine + Metoprolol Succinate for the specified duration; (4) Complete clinical data (baseline characteristics, follow-up records, and outcome assessments). Exclusion criteria: (1) Severe hepatic/renal insufficiency; (2) Allergy to study drugs; (3) Severe mental/cognitive impairment; (4) Other serious diseases affecting HF evaluation (e.g., advanced malignancies).

### Patient screening and selection bias assessment

**Figure 1:**
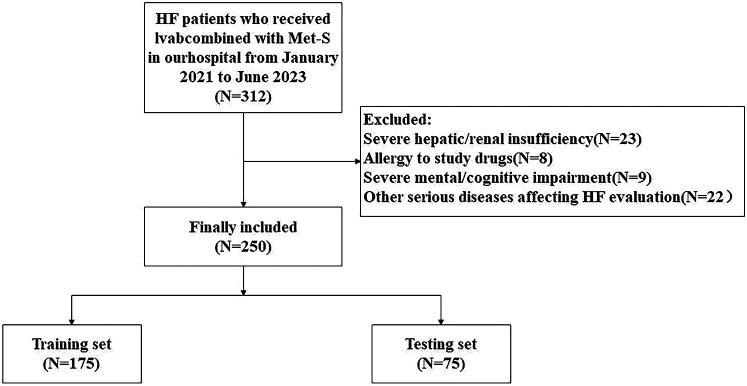
Study flow diagram: patient screening, enrollment, and allocation.

Initially, 312 HF patients were screened. After exclusions (*n* = 62), as shown in Figure 1. 250 were enrolled and randomized into training (*n* = 175) and verification (*n* = 75) sets. Baseline characteristics showed no significant differences between included/excluded patients (all *P* > 0.05) (Table 1**)**, indicating minimal selection bias.

**Table 1:** Comparison of baseline characteristics between included and excluded patients.

Characteristic		Included (*n* = 250)	Excluded (*n* = 62)	Statistical values	*P* Value
Age (years)		62.82 ± 7.14	63.58 ± 7.39	0.745	0.456
Gender	Male	133 (53.20)	30 (48.39)	0.461	0.497
	Female	117 (46.80)	32 (51.61)		
LVEF (%)		42.01 ± 6.32	40.83 ± 6.57	1.305	0.192
BNP (pg/ml)		342.54 ± 80.87	350.29 ± 85.61	0.667	0.504

### Sensitivity analysis for missing data

To assess the robustness of our findings, we conducted a sensitivity analysis for patients with partially missing data (*n* = 12). These patients had missing values in ≤10% of baseline or outcome variables. Using multiple imputation (MI) with chained equations (MICE) in R software, we generated five imputed datasets. The analysis revealed no significant differences in key outcomes (treatment efficacy, LVEF, BNP levels) between the imputed and complete-case datasets (all *P* > 0.05), as shown in Table 2. This suggests that the missing data were unlikely to bias our primary conclusions.

**Table 2:** Sensitivity analysis comparing complete-case and imputed datasets.

Characteristic	Complete-case (*n* = 238)	Imputed data (*n* = 250)	Statistical values	*P* value
Treatment Efficacy (%)	186 (78.15)	194 (77.60)	0.021	0.883
LVEF (%)	42.13 ± 6.31	41.92 ± 6.37	0.365	0.714
BNP (pg/ml)	341.52 ± 80.87	343.21 ± 81.12	0.230	0.817

### Background medical therapy

All enrolled patients received standard guideline-directed medical therapy for HFrEF. This included angiotensin converting enzyme inhibitor (ACEI), angiotensin II receptor blockers (ARB), or angiotensin receptor-neprilysin inhibitors (ARNi), as well as mineralocorticoid receptor antagonists (MRA), unless contraindicated or not tolerated. The proportion of patients receiving ACEI/ARB/ARNi was 98.4% (246/250), a detailed breakdown of the specific agent usage was as follows: ACEI 62.8% (157/250), ARB 24.8% (62/250), ARNi 10.8% (27/250). The proportion receiving MRA was 95.2% (238/250). There were no significant differences in the usage rates of ACEI/ARB/ARNi between the training and testing sets (Training set: 98.3% [172/175]; Testing set: 98.7% [74/75]), MRA (Training set: 94.9% [166/175]; Testing set: 96.0% [72/75]), and the individual agents between the training and testing sets (Training set: ACEI 63.4% [111/175], ARB 24.6% [43/175], ARNi 10.3% [18/175]; Testing set: ACEI 61.3% [46/75], ARB 25.3% [19/75], ARNi 12.0% [9/75]; all *P* > 0.05). Similarly, no significant differences were found between the effective and ineffective groups in the training set for usage rates of ACEI/ARB/ARNi (Effective: 98.5% [133/135]; Ineffective: 97.5% [39/40]), MRA (Effective: 96.3% [130/135]; Ineffective: 90.0% [36/40]), or the individual agents (Effective group: ACEI 64.4% [87/135], ARB 23.7% [32/135], ARNi 10.4% [14/135]; Ineffective group: ACEI 60.0% [24/40], ARB 27.5% [11/40], ARNi 10.0% [4/40]; all *P* > 0.05).

Loop diuretics were administered to 92.0% (230/250) of patients to manage fluid overload. The specific type (e.g., furosemide, torasemide) and dose of diuretics were individualized based on each patient's clinical status, signs of congestion, and renal function, with the goal of achieving and maintaining euvolemia. Consequently, the daily doses varied considerably and were frequently adjusted during follow-up. The mean daily loop diuretic dose (in furosemide equivalents) at the end of the study was comparable between the training and testing sets (Training set: 42.5 ± 18.3 mg; Testing set: 40.8 ± 17.6 mg; *P* = 0.512) and between the effective and ineffective groups in the training set (Effective group: 41.2 ± 17.1 mg; Ineffective group: 45.7 ± 19.8 mg; *P* = 0.187), indicating that diuretic therapy was aggressively optimized for all patients as per standard care and the difference in clinical efficacy was not attributable to differences in diuretic dosing.

### Treatment of IVAB combined with Met-S

The initial dose of Met-S was determined according to the severity and tolerance of heart failure in patients, following current international guidelines. For patients with cardiac function classification II, the initial dose was 47.5 mg once daily. For patients with cardiac function III–IV, the initial dose was 11.875 mg or 23.75 mg once daily, followed by gradual increases in dose as tolerated. Ivabradine was initiated only after the target or maximally tolerated dose of Met-S was achieved, as per evidence-based recommendations. The initial dose of Ivab was 5 mg twice daily, adjusted based on heart rate response and tolerability, with a maximum dose of 7.5 mg twice daily. Dose adjustments were made every 2–4 weeks, ensuring heart rate remained above 50 beats per minute and systolic blood pressure was maintained above 85 mmHg. Treatment duration was six months.

### Inclusion of indicators

Patients' basic information (age, gender, BMI, smoking history, drinking history), and medical history (hypertension, diabetes, coronary heart disease, atrial fibrillation, valvular heart disease) were investigated by questionnaire before treatment.

The left ventricular ejection fraction (LVEF), the left ventricular end diastolic diameter (LVEDD), and the right ventricular ejection fraction (RVEF) were all examined by echocardiography, and cardiac images were obtained by an ultrasonic probe. The LVEF and RVEF examinations were performed using software to analyze the volume changes during ventricular contraction and relaxation, and calculate the ejection fraction. LVEDD is the largest inner diameter of the measured left ventricle at end diastolic.

Systolic and diastolic blood pressure were measured use a mercury sphygmomanometer. The ECG machine records the electrical activity of the heart and accurately calculates the number of heart beats per minute. The 6-minute walk test (6 MWT) required the patient to walk in a flat, accessible corridor as fast as possible within 6 min, and the walking distance was measured, timed and recorded by a dedicated person.

The venous blood was drawn out on an empty stomach of the patient. The contents of creatinine and urea nitrogen as well as the concentrations of potassium and sodium ions in the blood were detected by a biochemical analyzer. The blood glucose concentration was measured by a blood glucose meter, and the brain natriuretic peptide (BNP) content in the blood was determined by a chemiluminescent immunoassay analyzer.

### Treatment effect judgment method

To evaluate the efficacy of ivabradine in combination with metoprolol succinate in patients with heart failure after six months of treatment, the following quantitative criteria were used:

Effective: Improvement in at least one of the following parameters compared to baseline: Improvement in cardiac functional classification by at least 1 grade compared to baseline, as assessed by the New York Heart Association (NYHA) classification. Reduction in the number of heart failure-related re-hospitalizations or emergency visits by ≥50% compared to the 6-month period prior to treatment.

Increase in 6-minute walk distance (6 MWT) by ≥30 m compared to baseline.

Reduction in BNP levels by ≥30% compared to baseline.

No effect:No improvement or worsening in NYHA functional class. No reduction or an increase in heart failure-related re-hospitalizations or emergency visits. No improvement or a decrease in 6 MWT distance (<30 m increase). No reduction or an increase in BNP levels (<30% reduction). Patient death during the treatment period.

Assessment method: All assessments were performed by two independent cardiologists blinded to the treatment groups. Discrepancies were resolved by a third senior cardiologist. The NYHA classification was determined using standardized criteria during clinical evaluation, and the 6 MWT was conducted in a controlled environment by trained personnel.

### Statistical analysis

To ensure robust internal validation, we performed: (1) 10-fold cross-validation repeated 5 times, which yielded mean AUC values of 0.848 (95% CI: 0.790–0.906) for the training set; (2) bootstrap resampling (1,000 iterations) with optimism correction, showing minimal optimism (0.012) in the C-index; and (3) split-sample validation (70:30 ratio). These complementary approaches confirmed the model's stability beyond simple AUC/C-index metrics. This prediction model study followed the TRIPOD (Transparent Reporting of a multivariable prediction model for Individual Prognosis or Diagnosis) statement guidelines for development and validation. All key elements including participant selection, predictor assessment, outcome determination, and analysis methods are reported accordingly. Model discrimination was assessed using C-index, with bootstrapping (1,000 resamples) to calculate confidence intervals. We employed both logistic regression and restricted cubic splines (RCS) with 3 knots to evaluate potential non-linear relationships between continuous predictors (LVEF, LVEDD, 6 MWT, heart rate, and BNP levels) and treatment outcomes. Interaction terms between key predictors were tested using multiplicative terms in the logistic model. The linearity assumption was verified through Martingale residual plots, and variance inflation factors (VIF) were calculated to assess multicollinearity. Model comparisons between linear and non-linear approaches were performed using likelihood ratio tests and Akaike Information Criterion (AIC). All data were analyzed using SPSS 26.0 and R software (R4.0.0). Measurement data of normal distribution, confirmed by the Shapiro–Wilk test, were expressed as mean ± standard deviation (SD). Non-normally distributed data were presented as median (interquartile range). For binary classification, the area under the receiver operating characteristic curve (AUC) was calculated to evaluate model discrimination. Calibration was assessed using calibration curves and the Hosmer-Lemeshow test. Decision curve analysis (DCA) was performed to evaluate clinical utility. Confidence intervals (CIs) for the AUC, sensitivity, specificity, and other metrics were calculated using bootstrapping with 1,000 resamples to ensure robust estimates. Logistic regression models were used to compute odds ratios (ORs) with 95% CIs derived from the standard errors of the regression coefficients. Note that in binary logistic regression, the C-index is mathematically equivalent to AUC; thus, only AUC values are reported here for clarity.

## Results

### Comparison of treatment inefficiency and clinical parameters between training set and validation set

Forty patients (22.85%) in the training set and 15 patients (20.00%) in the testing set were ineffective. Among the effective groups, 135 patients (77.14%) in the training set and 60 patients (80.00%) in the testing set showed good clinical outcomes. There was no significant difference in the treatment efficiency or clinical parameters between the training set and the testing set (*P* > 0.05) (Table 3). There was no significant difference in the final daily doses of metoprolol succinate (Met-S) (Ineffective: 78.6 ± 25.4 mg vs. Effective: 82.3 ± 23.1 mg, *P* = 0.412) or ivabradine (Ivab) (Ineffective: 8.9 ± 2.1 mg vs. Effective: 9.2 ± 1.8 mg, *P* = 0.385) between the ineffective and effective groups in the training set. This indicates that the difference in clinical efficacy was not attributable to differences in the achieved doses of the study drugs.

**Table 3:** Treatment ineffectiveness, clinical parameters, and other related parameters between training Set and testing Set.

Project		Training set (*n* = 175)	Testing set (*n* = 75)	Statistical values	*P* value
Age (years)		62.96 ± 7.02	62.51 ± 7.20	0.464	0.643
Gender	Male	95 (54.29)	38 (50.67)	0.276	0.599
	Female	80 (45.71)	37 (49.33)		
BMI (kg/m^2^)		23.60 ± 2.63	23.21 ± 2.56	1.088	0.278
Smoking history	Yes	52 (29.71)	20 (26.67)	0.238	0.626
	No	123 (70.29)	55 (73.33)		
Drinking history	Yes	83 (47.43)	32 (42.67)	0.479	0.489
	No	92 (52.57)	43 (57.33)		
History of hypertension	Yes	67 (38.29)	26 (34.67)	0.294	0.587
	No	108 (61.71)	49 (65.33)		
History of diabetes	Yes	42 (24.00)	16 (21.33)	0.210	0.647
	No	133 (76.00)	59 (78.67)		
History of coronary heart disease	Yes	65 (37.14)	25 (33.33)	0.331	0.565
	No	110 (62.86)	50 (66.67)		
History of atrial fibrillation	Yes	59 (33.71)	23 (30.67)	0.182	0.670
	No	116 (66.29)	52 (69.33)		
History of valvular heart disease	Yes	26 (14.86)	10 (13.33)	0.099	0.753
	No	149 (85.14)	65 (86.67)		
LVEF (%)		42.13 ± 6.42	41.82 ± 6.23	0.348	0.728
LVEDD (mm)		56.96 ± 6.84	57.05 ± 6.62	0.087	0.931
RVEF (%)		47.90 ± 5.70	46.45 ± 5.39	1.885	0.061
6 MWT (m)		319.53 ± 51.34	315.20 ± 50.80	0.612	0.541
Systolic blood pressure (mmHg)		130.16 ± 10.69	129.81 ± 10.50	0.235	0.814
Diastolic blood pressure (mmHg)		79.95 ± 7.48	78.24 ± 6.98	1.690	0.092
Heart rate (beats/min)		73.38 ± 9.10	72.69 ± 8.85	0.557	0.578
Blood creatinine (mol/L)		97.87 ± 13.72	98.31 ± 12.35	0.243	0.809
Urea nitrogen level (mmol/L)		5.90 ± 1.10	5.80 ± 1.05	0.611	0.542
Blood Potassium Levels (mmol/L)		4.25 ± 0.53	4.27 ± 0.67	0.298	0.766
Blood sodium level (mmol/L)		139.36 ± 1.91	139.07 ± 1.88	1.140	0.255
Fasting glucose level (mmol/L)		6.06 ± 1.38	6.25 ± 1.34	1.030	0.304
Glycated hemoglobin level (%)		7.05 ± 0.88	7.12 ± 0.94	0.637	0.524
BNP levels (pg/ml)		343.11 ± 81.41	339.25 ± 80.25	0.344	0.731

### Comparison of clinical parameters between ineffective and effective groups in the training set

In the training set, the results of univariate analysis showed that the history of atrial fibrillation, valvular heart disease, LVEF, LVEDD, 6 MWT, systolic blood pressure, diastolic blood pressure, heart rate, blood creatinine, urea nitrogen level, and BNP level between the treatment ineffective group (*n* = 40) and the effective group (*n* = 135) had statistical differences (*P* < 0.05). as shown in Table 4.

**Table 4:** Comparison of clinical parameters between ineffective and effective groups in the training set.

Project		Invalid group (*n* = 40)	Valid group (*n* = 135)	Statistical values	*P* Value
Age (years)		63.50 ± 7.60	62.80 ± 7.00	0.553	0.581
Gender	Male	23 (57.50)	72 (53.33)	0.216	0.642
	Female	17 (42.50)	63 (46.67)		
BMI (kg/m^2^)		23.81 ± 3.02	23.54 ± 2.51	0.569	0.570
Smoking history	Yes	14 (35.00)	38 (28.15)	0.694	0.405
	No	26 (65.00)	97 (71.85)		
Drinking history	Yes	19 (47.50)	64 (47.41)	0.001	0.992
	No	21 (52.50)	71 (52.59)		
History of hypertension	Yes	14 (35.00)	53 (39.26)	0.237	0.626
	No	26 (65.00)	82 (60.74)		
History of diabetes	Yes	10 (25.00)	32 (23.70)	0.028	0.866
	No	30 (75.00)	103 (76.30)		
History of coronary heart disease	Yes	13 (32.50)	52 (38.52)	0.479	0.489
	No	27 (67.50)	83 (61.48)		
History of atrial fibrillation	Yes	17 (42.50)	32 (23.70)	5.408	0.020
	No	23 (57.50)	103 (76.30)		
History of valvular heart disease	Yes	10 (25.00)	16 (11.85)	4.217	0.040
	No	30 (75.00)	119 (88.15)		
LVEF (%)		38.51 ± 5.80	43.21 ± 6.21	4.264	0.001
LVEDD (mm)		60.24 ± 7.00	56.00 ± 6.50	3.557	0.001
RVEF (%)		46.75 ± 6.21	48.24 ± 5.51	1.458	0.147
6 MWT (m)		300.25 ± 50.20	325.24 ± 50.44	2.756	0.006
Systolic blood pressure (mmHg)		133.22 ± 11.50	129.25 ± 10.31	2.078	0.039
Diastolic blood pressure (mmHg)		82.35 ± 8.52	79.24 ± 7.02	2.340	0.020
Heart rate (beats/min)		79.54 ± 9.02	71.56 ± 8.31	5.229	0.001
Blood creatinine (µmol/L)		102.24 ± 14.52	96.58 ± 13.25	2.321	0.021
Urea nitrogen level (mmol/L)		6.31 ± 1.15	5.78 ± 1.05	2.732	0.007
Blood Potassium Levels (mmol/L)		4.21 ± 0.65	4.26 ± 0.51	0.630	0.530
Blood sodium level (mmol/L)		138.75 ± 2.52	139.54 ± 1.65	1.864	0.068
Fasting glucose level (mmol/L)		6.41 ± 1.55	5.96 ± 1.31	1.865	0.064
Glycated hemoglobin level (%)		7.29 ± 0.95	6.98 ± 0.85	1.977	0.050
BNP levels (pg/ml)		358.25 ± 81.34	335.65 ± 80.23	2.250	0.026

### Analysis of risk factors for clinical effects of IVAB combined with Met-S in the treatment of HF

Clinical effect was taken as the dependent variable (0 = effective [*n* = 135], 1 = ineffective [*n* = 40]), and the factor with *P* < 0.05 in single factor analysis was taken as the covariate. Further multivariate Logistic regression analysis showed that LVEF, LVEDD, 6 MWT, heart rate, and BNP level were the independent risk factors for the unsatisfactory clinical effects of Ivab combined with Met-S in the treatment of HF (*P* < 0.05), as shown in Table 5. RCS analysis revealed a non-linear relationship between BNP levels and treatment efficacy (P for non-linearity = 0.021), with a steeper increase in treatment failure risk above 400 pg/ml. A significant interaction was observed between LVEF and heart rate (*P* = 0.032), where the adverse effect of tachycardia was more pronounced in patients with LVEF < 35%. The linear logistic model (AIC = 142.6) showed comparable performance to the RCS-enhanced model (AIC = 140.2, *P* = 0.083 by likelihood ratio test), supporting our primary analysis while identifying threshold effects.

**Table 5:** Logistic regression analysis of risk factors for clinical effects.

Factor	B	S.E.	Wald	*P*	OR	95% CI
History of atrial fibrillation	0.475	0.689	0.476	0.490	1.609	0.417–6.207
History of valvular heart disease	0.070	0.855	0.007	0.935	1.072	0.201–5.724
LVEF	−0.130	0.042	9.479	0.002	0.878	0.809–0.954
LVEDD	0.081	0.040	4.240	0.039	1.085	1.004–1.172
6 MWT	−0.010	0.005	3.979	0.046	0.990	0.980–1.000
Systolic pressure	0.032	0.024	1.727	0.189	1.033	0.984–1.083
Diastolic pressure	0.063	0.035	3.340	0.068	1.065	0.995–1.140
Heart rate	0.106	0.028	14.351	0.001	1.112	1.052–1.174
Serum creatinine	0.026	0.019	1.940	0.164	1.027	0.989–1.066
Urea nitrogen level	0.137	0.245	0.315	0.574	1.147	0.710–1.853
BNP levels	0.008	0.003	5.637	0.018	1.008	1.001–1.014
Constant	−27.018	6.667	9.939	0.002		

### Establishment of nomogram prediction model for the clinical effect of IVAB combined with Met-S in the treatment of HF

**Figure 2:**
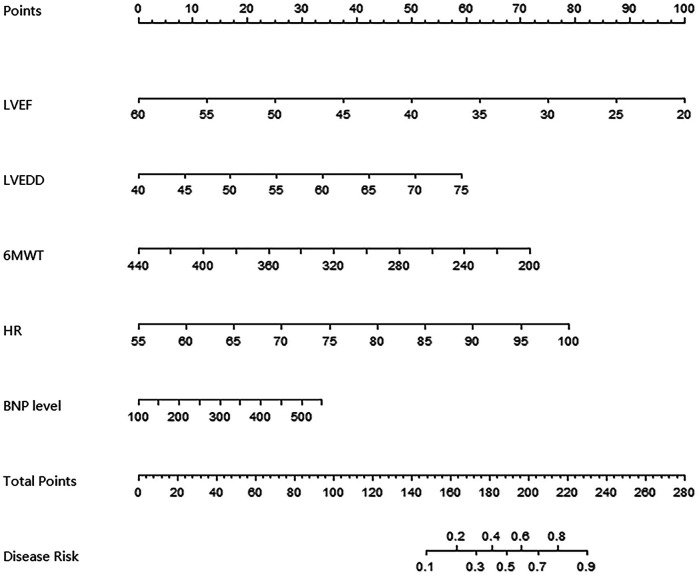
Alignment diagram of clinical effect prediction model of ivab combined with Met-S in the treatment of HF.

To capture potential non-linear relationships between predictors and outcomes, we explored alternative modeling approaches alongside the primary logistic regression model. Specifically, we evaluated a generalized additive model (GAM) with smoothing splines for continuous variables (LVEF, LVEDD, 6 MWT, heart rate, and BNP levels) to assess non-linearity. The GAM analysis revealed no significant deviations from linearity (all *P*-values for non-linear terms >0.05), supporting the use of linear assumptions in our final logistic regression model. Based on the independent risk factors identified by multivariate Logistic regression analysis, a nomogram prediction model for clinical effect of treatment was constructed. Each independent risk factor in the model was scored, and the total score for predicting clinical effect of treatment was calculated, which was reflected in the rate of poor clinical effect prediction. The higher the total score was, the higher the accuracy was in predicting poor clinical effect of treatment, as shown in Figure 2.

### Evaluation and validation of nomogram prediction model with poor clinical effect

**Figure 3:**
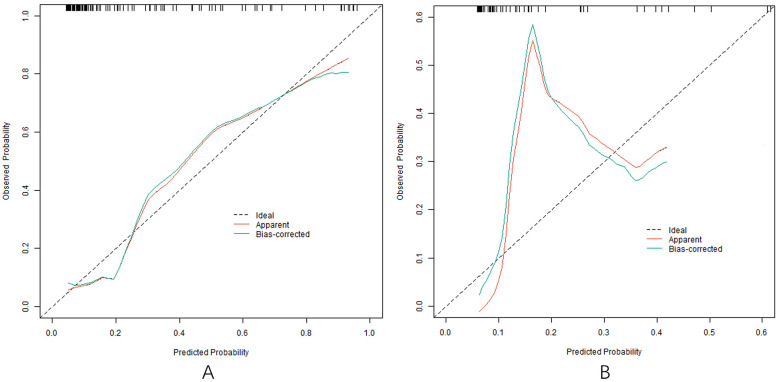
Calibration curve in the training set **(A)** and the testing set **(B)**.

**Figure 4:**
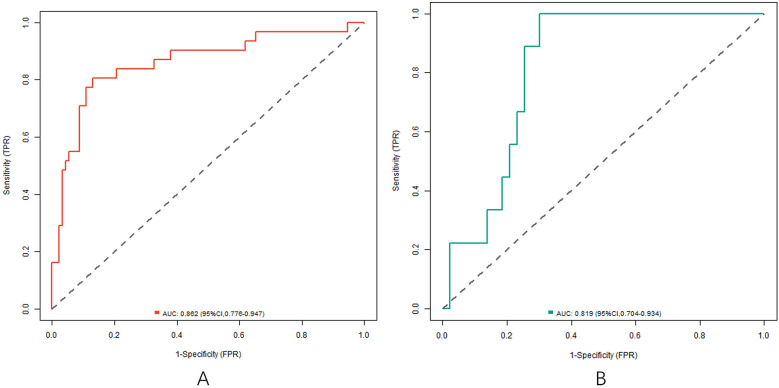
ROC curve in the training set **(A)** and the testing set **(B)**.

In the training and testing sets, the AUCs were 0.862 (95% CI: 0.776–0.947) and 0.819 (95% CI: 0.704–0.934), respectively, indicating excellent discrimination for the nomogram model predicting poor clinical efficacy. The sensitivity was 0.806 (95% CI: 0.712–0.900) and 0.750 (95% CI: 0.621–0.879), and the specificity was 0.870 (95% CI: 0.801–0.939) and 0.791 (95% CI: 0.676–0.906) for the training and testing sets, respectively. The calibration curve, assessed using bootstrapping (1,000 resamples), showed the mean absolute errors of predicted and actual values were 0.118 (95% CI: 0.092–0.144) and 0.139 (95% CI: 0.110–0.168), respectively. The Hosmer-Lemeshow test results were *χ*^2^ = 8.3042, *P* = 0.4043 (training set) and *χ*^2^ = 10.777, *P* = 0.2147 (testing set), demonstrating good model fit. Recalibration adjustments were applied to further optimize the model's performance. The calibration curves are shown in Figure 3 and the ROC curves are shown in Figure 4.

### Analysis of decision curve of nomogram prediction model of clinical effect of IVAB combined with Met-S in the treatment of HF

**Figure 5:**
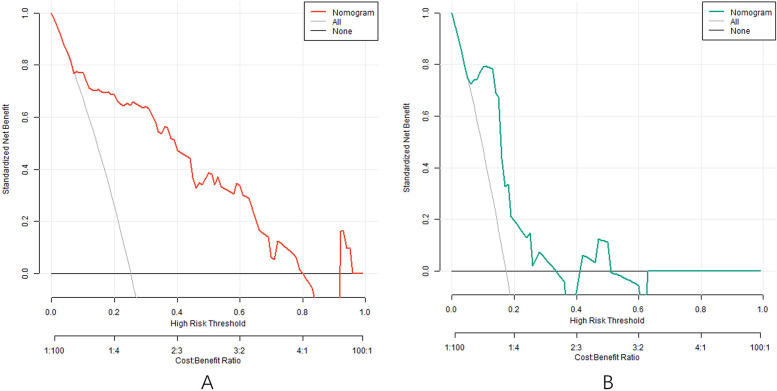
Decision curve in the training set **(A)** and the testing set **(B)**.

The decision curve analysis (DCA) evaluates the clinical utility of the nomogram model by quantifying net benefits across different threshold probabilities (*X*-axis: “cost-benefit” ratio). As shown in Figure 5, when the threshold probability ranges between 0.05 and 0.95, the nomogram model (red line) provides higher net benefits compared to the “treat-all” (blue line) or “treat-none” (gray line) strategies. This indicates that the model has significant clinical value in guiding individualized treatment decisions for HF patients receiving Ivab and Met-S therapy. For instance, at a threshold probability of 0.2 (where clinicians consider a 20% risk of treatment inefficacy acceptable), the model yields a net benefit of 0.35, demonstrating its superiority over empirical approaches.

## Discussion

In this study, we adhered to evidence-based protocols by initiating beta-blocker therapy (Met-S) before introducing ivabradine, consistent with international guidelines. This approach ensures that patients first achieve the proven benefits of beta-blockade, which forms the cornerstone of HFrEF treatment. Ivabradine was subsequently added for patients who remained symptomatic with elevated heart rates despite optimal beta-blocker therapy. This sequential strategy aligns with the SHIFT trial protocol and current clinical practice in the US and EU, where ivabradine is indicated only after beta-blocker optimization. Our results demonstrate that this method is both safe and effective in the studied population. In the field of HF treatment, traditional therapeutic drugs such as diuretics, angiotensin converting enzyme inhibitor (ACEI) or angiotensin II receptor antagonist (ARB) have improved the symptoms and prognosis of patients to a certain extent, but still have many limitations (16, 17). Some patients have poor tolerance to these drugs, and as the disease progresses, it is difficult to meet the demand for treatment with traditional drugs alone. The combination of Ivab and Met-S offers new promise for the treatment of HF due to the theoretically synergistic effects. It is of great clinical significance to deeply analyze the factors affecting the therapeutic effect and establish an effective prediction model. Our findings align with recent studies demonstrating the critical role of cardiac remodeling markers in treatment response. The identified predictors (LVEF, LVEDD, 6 MWT, HR, BNP) collectively reflect both structural and functional aspects of heart failure pathophysiology. The particularly strong predictive value of BNP levels above 400 pg/ml corroborates findings from Otsuka et al, suggesting this biomarker may serve as a key threshold for therapeutic decision-making.

In this study, through univariate and multivariate analysis, we screened out the relevant factors that affect the clinical effect of Ivab combined with Met-S in patients with HF. The results showed that LVEF, LVEDD, 6 MWT, heart rate and BNP level were independent risk factors for clinical effects. LVEF reflects the blood pump function of the heart. The lower LVEF indicates that the myocardial contractility is weaker, and the blood pump ability of the heart is poorer, which will directly affect the therapeutic effect (18). The enlargement of LVEDD often indicates the left ventricular dilatation, changes in cardiac structure and aggravation of myocardial remodeling, which in turn affects the normal function of the heart and leads to poor therapeutic effect (19). The distance of 6 MWT reflects the patient's exercise tolerance and cardiopulmonary function reserve. The shorter the distance, the poorer the patient's cardiopulmonary function will be and the reaction to treatment may be poorer (20). Heart rate is one of the important indicators of cardiac function. Too fast heart rate will increase myocardial oxygen consumption, aggravate the heart burden and affect the therapeutic effect (21). The BNP level is closely related to the severity of heart failure. The higher the BNP level is, the more severe the heart failure will become, and the difficulty in treatment will correspondingly increase (22). While LVEF, LVEDD, 6 MWT, heart rate and BNP are established prognostic markers in HF, our study specifically demonstrates their predictive value for treatment response to Ivab-Met-S combination therapy. The interaction between LVEF and heart rate (*P* = 0.032) and the non-linear relationship with BNP (threshold effect at 400 pg/ml) provide novel insights into how these conventional parameters specifically influence this drug combination's efficacy.

Based on the independent risk factors identified by multivariate Logistic regression analysis, the nomogram prediction model was constructed in this study. The model had good discrimination, and the C-index index in the training set and the testing set reached 0.856 and 0.816, respectively, indicating that the model could distinguish between patients with effective treatment and patients with ineffective treatment. The calibration curve showed that the predicted values were in good agreement with the actual values, and the Hosmer Lemeshow test result also showed that the model had good goodness of fit. Meanwhile, the AUCs were 0.862 and 0.819 in the training set and testing set, respectively, further verifying the accuracy and reliability of the model. The analysis of decision curve showed that when the threshold probability was about 0.05–0.95, the decision on the clinical effect of Ivab combined with Met-S in the treatment of HF predicted by the nomogram model constructed in this study had more clinical benefits, indicating that the model had high clinical application value.

Compared to established HF risk scores like MAGGIC or Seattle Heart Failure Model, our nomogram provides specific prediction for Ivab-Met-S response rather than general prognosis. While these tools predict mortality/hospitalization, our model focuses on therapeutic efficacy—a distinct clinical question requiring different predictors and validation approaches as outlined in TRIPOD guidelines. The nomogram's risk strata (low <30, intermediate 30–60, high >60) align with current HF management tiers, allowing seamless integration into clinical workflows. For high-risk patients, we suggest: (a) intensifying monitoring to biweekly visits with NT-proBNP tracking, (b) considering early addition of SGLT2 inhibitors per 2022 AHA/ACC/HFSA guidelines, and (c) addressing modifiable risk factors like fluid status optimization. Although our model demonstrated good discrimination and calibration, the relatively limited sample size (*n* = 250) raises potential concerns about overfitting. We mitigated this risk by: (1) restricting candidate predictors to 5 clinically meaningful variables (LVEF, LVEDD, 6 MWT, heart rate, BNP) based on prior evidence, maintaining an events-per-variable ratio >15; (2) using regularization techniques in the logistic regression; and (3) performing external validation in an independent set (*n* = 75). However, the model's performance should be further validated in larger, multicenter cohorts. However, this study also has certain limitations. First, although a number of factors that may influence the therapeutic effect have been included, some potential influencing factors may still be omitted. In addition, due to current limitations of research resources and conditions, study samples were only obtained from our hospital without external verification, so the representation of samples was relatively limited. Prospective, multi-center large-scale research can be considered for future research to further verify the results of this study and include more factors for analysis, so as to continuously improve the prediction model. At the same time, new technologies such as gene detection can be combined to deeply explore the mechanisms affecting the therapeutic effects at the molecular level, so as to provide a more solid theoretical basis for the personalized treatment of HF. Our sensitivity analysis using alternative modeling approaches (e.g., GAM) confirmed that linear relationships adequately captured the associations between key predictors (LVEF, LVEDD, 6 MWT, heart rate, BNP) and treatment efficacy. While non-linear effects were not statistically significant in this cohort, future studies with larger samples may benefit from machine learning techniques (e.g., random forests or gradient boosting) to uncover complex interactions.

In summary, LVEF, LVEDD, 6 MWT, heart rate, and BNP levels affect the clinical efficacy of Ivab in combination with Met-S in patients with HF. The nomogram prediction model constructed in this study had high accuracy and clinical application value, which could provide a reference for clinicians to predict the treatment effect of patients, help to develop a more reasonable treatment plan, and improve the treatment effect and quality of life of patients with HF. However, there are still some limitations in this study and further research is needed to improve it.

## Data Availability

The raw data supporting the conclusions of this article will be made available by the authors, without undue reservation.
